# Lateralization of Hippocampal Dentate Spikes and Sharp‐Wave Ripples in Urethane Anesthetized Rats Depends on Cholinergic Tone

**DOI:** 10.1002/hipo.70035

**Published:** 2025-09-23

**Authors:** Miriam S. Nokia, Sanna Lensu, Suvi‐Maaria Lehtonen, Tero Harjupatana, Markku Penttonen

**Affiliations:** ^1^ Department of Psychology and Centre for Interdisciplinary Brain Research University of Jyväskylä Jyväskylä Finland; ^2^ Department of Physics, Nanoscience Center University of Jyväskylä Jyväskylä Finland

**Keywords:** autonomic nervous system, breathing, hippocampus, oscillations, phase synchrony

## Abstract

Neural activity and bodily functions are inherently rhythmic and related to each other. The occurrence of hippocampal sharp‐wave ripples (SPW‐Rs) and dentate spikes (DSs) supporting memory consolidation is regulated by the overall state of the brain, and they also seem to aggregate to a certain phase of the breathing cycle in naturally sleeping mice. Further, SPW‐Rs and DSs synchronize to a variable degree between hemispheres, but how this is affected by the neural and bodily state is unclear. Here, we recorded dorsal hippocampal local‐field potentials, electrocardiogram, and respiration for several hours under urethane anesthesia in adult male Sprague–Dawley rats. For a subset of rats, we injected atropine (50 mg/kg, i.p.) halfway into the recording to decrease cholinergic and parasympathetic tone. We found variable relation of hippocampal oscillations to breathing phase and none to the cardiac cycle phase. A decrease in breathing rate implying increased parasympathetic tone preceded the start of SPW‐R bouts. Roughly 90% of DSs and half of SPW‐Rs were unilateral. In most rats, SPW‐Rs were more often bilateral during slow breathing compared to faster breathing. Atropine reduced the proportion of bilateral SPW‐Rs. Both nonrapid eye movement sleep‐like state and atropine increased the proportion of bilateral DSs under urethane anesthesia. Finally, in naturally sleeping rats, both DSs and SPW‐Rs were bilateral ~60% of the time. In sum, urethane seems to desynchronize DSs but not SPW‐Rs, and a low cholinergic and/or parasympathetic tone seems to dissociate SPW‐Rs and to synchronize DSs in the two hippocampi. Whether these findings have relevance in terms of memory consolidation and behavior should be investigated in the future.

## 1 Introduction

Hippocampus is crucial for episodic memory formation. Efficient memory consolidation during sleep and rest relies at least in part on hippocampal sharp‐wave ripples (SPW‐Rs) (rats: [Girardeau et al. 2009], rabbits: [Nokia et al. 2012]) and dentate spikes (DSs) (Farrell et al. 2024; Lensu et al. 2019; Nokia et al. 2017). SPW‐Rs are high‐amplitude, fast (> 120 Hz) rhythmic events in the hippocampal CA1, during which neural activation patterns representing awake experiences are replayed in the cortico‐hippocampal loop (Buzsaki 2015). DSs originate in the entorhinal cortex (EC) and are evident in the hilus of the dentate gyrus (DG) (Bragin, Jando, Nadasdy, van Landeghem, et al. 1995; Penttonen et al. 1997) and might also be related to memory replay at the level of neural assemblies (McHugh et al. 2024).

Both SPW‐Rs and DSs are more frequent during nonrapid eye movement sleep (NREM) compared to rapid eye movement sleep (REM). During NREM and states resembling it, the hippocampal cholinergic tone is low (Zhang et al. 2021), theta oscillations (3–12 Hz) are weak (Buzsaki 2002; Kramis et al. 1975; Stewart and Vanderwolf 1987), and respiration is slow (Pagliardini et al. 2012). Interestingly, respiration synchronizes with the rhythmic electrophysiological activity of the brain at large (Klimesch 2018; Kluger and Gross 2021), and in naturally sleeping mice, SPW‐Rs and DSs are time‐locked to a certain phase of breathing (Karalis and Sirota 2022; Liu et al. 2017). Thus, both neural and bodily states affect SPW‐R and DS occurrence.

Originally, it was reported that DSs are mainly bilateral in natural sleep and rest (Bragin, Jando, Nadasdy, van Landeghem, et al. 1995). However, according to our recent observation in urethane‐anesthetized rats, DSs most often take place independently in the left or right hippocampus (Lehtonen et al. 2022). SPW‐Rs, on the other hand, might be more bilateral in nature ([Hernández‐Recio et al. 2023] but see also: [Villalobos et al. 2017]). In line, detailed analysis of field‐potential generation under urethane anesthesia during nontheta states suggests globally highly correlated activity in the CA1 (i.e., simultaneous SPWs, yet desynchrony at the level of ripples), but largely uncorrelated activity in the left and right DG, especially in response to lateral EC input (Hernández‐Recio et al. 2023). As for now, it is not clear how the level of synchrony between the right and left hippocampus regarding SPW‐Rs and DSs fluctuates across neural or bodily states, and whether anesthetics such as urethane influence this.

To study the interhemispheric synchrony and connections of hippocampal oscillatory events to bodily rhythms, we recorded hippocampal local‐field potentials (LFPs), electrocardiogram (ECG), and respiration in urethane‐anesthetized male adult Sprague–Dawley rats. We expected to find an association of respiration phase (mice: [Karalis and Sirota 2022]) and rate (Pagliardini et al. 2012) with the occurrence of SPW‐Rs and DSs. Further, we explored the timing of SPW‐Rs and DSs in the left vs. right hippocampus, expecting to possibly detect an effect of overall neural (REM‐like vs. NREM‐like) and bodily (slow vs. faster breathing) state. We also studied the effect of cholinergic/parasympathetic tone on the above‐mentioned phenomena using atropine (50 mg/kg, i.p.). We expected a decrease in hippocampal theta (Kramis et al. 1975; Stewart and Vanderwolf 1987), in respiration rate (Pagliardini et al. 2012), and in the interhemispheric synchrony of hippocampal oscillations (Gedankien et al. 2023). In addition, we conducted LFP recordings in a separate group of freely moving adult female rats in natural sleep and awake rest to compare the level of interhemispheric synchrony of SPW‐Rs and DSs to that observed during urethane‐anesthesia.

## 2 Methods

### 2.1 Ethics Statement

The ARRIVE guidelines (https://arriveguidelines.org/arrive‐guidelines) were followed. All experimental procedures were approved by the Regional State Administrative Agency of Southern Finland and implemented in accordance with Directive 2010/63/EU.

### 2.2 Subjects

The subjects used in the acute recordings under anesthesia were 17 adult male Sprague–Dawley rats (Envigo, Netherlands). In the chronic awake rest and natural sleep recordings, we used six adult female Sprague–Dawley rats. Animals were housed in groups or pairs at the University of Jyväskylä Laboratory center until surgery. All rats were kept in Macrolon 1354, type IV cages (Techniplast, Buguggiate, Italy) and had aspen chips (Tapvei, Kaavi, Finland) as bedding material and a red plastic tube as a toy and shelter. After surgery, the rats used for awake and sleep recordings were housed alone, in pet rodent cages (55 × 39 × h 27 cm, Ferplast Mini Duna Multy, Italy), until the end of the experiment. Rats were maintained on a 12–12 h light–dark cycle, with lights on at 08:00 a.m. Food and water were freely available, and room temperature and humidity were controlled at 21°C ± 2°C and 50% ± 10%, respectively. All procedures were conducted during the light portion of the cycle. Rats were handled before experimental use.

### 2.3 Surgery

#### 2.3.1 Acute Experiment Under Urethane Anesthesia

Rats were weighed (mean, M [standard deviation, SD]: 352 [31] g) and then anesthetized with urethane (dose: 1.3 g/kg, concentration: 0.24 g/mL diluted in sterile water, injection route: i.p.). Once anesthetized, the rat was placed on top of a heating pad set at 37°C. The head of the animal was attached to a stereotaxic instrument (David Kopf Instruments, Model 962, Tujunga, CA, US). A piezo sensor was placed under the rat's ribcage to record respiration, and two 23‐G needle‐electrodes were placed under the skin, one on each side, to record ECG. Under local anesthesia (bupivacaine: 1 mL, 2.5 mg/mL, or lidocaine: 0.5 mL, 20 mg/mL, s.c.), a cut was made to the scalp with a scalpel, and excess skin was removed. Then, craniotomies were drilled over the left and right dorsal hippocampus (3.5–5.0 mm posterior, 1–2.5 mm lateral to the bregma). Two 32‐electrode linear silicon probes with 65 μm between electrode contacts (E32‐65‐S1‐L6‐NT, Atlas Neuroengineering, Leuven, Belgium) were implanted in the dorsal hippocampus, one in each hemisphere, targeting the tip at the lower blade of the DG at ~3.6 mm below dura, 1.5–2 mm lateral, and 3.8 mm posterior to bregma (see Figure S1 for an example of probe placement and raw signal). A 27 G injection needle inserted into the cerebellum served as the reference, while another needle inserted into the neck muscles served as ground. All rats were hydrated with s.c. injections of 0.9% NaCl every few hours.

#### 2.3.2 Chronic Experiment

Rats were weighed (274 [11] g) and then injected with saline and anti‐inflammatory analgesics (carprofen 10 mg/kg, 10 mg/mL, s.c. and dexamethasone 0.2 mg/kg, 0.04 mg/mL, s.c.). Then, anesthesia was induced with 5% and maintained at 1%–2.5% isoflurane using an air flow rate of ~0.5 L/min. Saline was injected (2 mL, s.c.) every hour and body temperature was maintained with a heating pad set at 37°C underneath the rat. The head was fixed to a stereotactic frame (Kopf Instruments, CA, US) and fur from the top of the head was shaved. After careful disinfection of the skin, an incision was made with a scalpel. The skull was cleaned with 3% H_2_O_2_ and holes were drilled for all the implants. Screws connected in pairs and used as ground (4 mm anterior to bregma, 2 mm lateral to midline) and reference (7 mm posterior to bregma, 3 mm lateral to midline) were implanted. Then, eight monopolar recording electrodes assembled in‐house from 50‐μm Formwar‐coated nichrome wire (762,000, A‐M Systems, Carlsborg, WA) aimed at the dorsal hippocampus were implanted at 3.3–4.2 mm posterior to bregma, 1.2–2.4 mm lateral to midline, and 2.6–4.0 mm below bregma. Two screws were implanted on the cerebellum at 10.5 mm posterior to bregma and 1.5 mm lateral to midline (not used in this experiment). The pins on the recording electrodes and those connected to the ground and reference screws were assembled to a 1.27 mm single‐row pin connector. Finally, the whole construction was cemented in place using UV‐curable dental glue and cold‐curing dental acrylic. Postoperative analgesia (carprofen 10 mg/kg, 10 mg/mL, s.c.) was continued for 5 days, and experiments started at the earliest a week from surgery.

### 2.4 Acute Recording

The room was kept quiet, and lighting dim throughout the recording. LFPs from the two probes were band‐pass filtered (1–5000 Hz) and stored (20‐kHz sampling rate) using Multichannel Systems (MCS, Reutlingen, Germany) equipment (μPA32 connected to a FA64I and finally to a USB‐ME64 unit). ECG and piezo signals were high‐pass filtered (0.1 Hz), amplified (ECG: 100×; piezo: 50×), and low‐pass filtered (ECG: 600 Hz; piezo: 2 Hz) (Axon Cyberamp 380, Molecular Devices Corporation, Union City, CA), fed to the analog input of the USB‐ME64, digitized, and stored along with the LFPs. Note that the lowpass filtering of the piezo signal imposed a 159 ms delay to the digitized, stored signal. This delay was considered in all analysis. Recordings lasted up to 6 h. All anesthetized rats were euthanized by decapitation.

#### 2.4.1 Atropine Injection

For a subset of rats (*n* = 8), atropine (Atropine sulfate [monohydrate] PHR1379, Sigma‐Aldrich, Steinheim, Germany) was administered i.p. (50 mg/kg, 5 mg/mL diluted in 0.9% NaCl) 3 h after starting the recording. For the rats not receiving atropine (*n* = 9), 2 mL of 0.9% NaCl was injected subcutaneously.

### 2.5 Chronic Recording

For recording LFPs during awake rest and sleep, rats with chronically implanted recording electrodes in the dorsal hippocampus were placed in a clear acrylic cylinder (diameter 20 cm, height 40 cm), inside a sound‐attenuated cabinet (ENV‐018 V, Med Associates, Fairfax, VT) lit dimly. A wireless 8‐ch headstage (W2100‐HS8, MCS, Reutlingen, Germany) was attached to the pin connector on the rat's head to obtain LFPs and accelerometer signals. The data were stored with the W2100‐System (MCS) at 5 kHz. The rat was allowed to move freely in the cylinder for ~2 h, and the recording was repeated several times per animal, with days or weeks in between recordings. Note that the quality of recordings is very stable with this type of chronic implant. Also, note that during daytime recordings, the rats mostly rest and sleep.

### 2.6 Verifying Electrode Locations

Location of the linear probe electrodes was determined based on signal properties and prior experience (Lehtonen et al. 2022). For further verification, in a group of rats from which data is not reported here, we prepared histological samples of the hippocampus to locate the tip of the electrode and to visualize the corresponding raw signals (see Figure S1).

The placement of home‐made monopolar electrodes in the hippocampi of chronically implanted rats was determined from structural images obtained with a microCT scan. In short, the rat was overdosed with pentobarbital (400 mg/mL, Euthasol Vet, diluted 1:2 in saline, 1 mL injected i.p.) and then perfused with 200 mL of 0.1 M PBS followed by 200 mL of 4% Formalin. Then, the brain was extracted and stored in 4% Formalin for 24 h. This was followed by a rising series of ethanol solutions from 20% to 90% (changed daily). At last, the brains were stored in 90% ethanol +1.5% iodine for 72 h (Chen et al. 2018). For imaging, the brain was embedded in paraffin wax inside a plastic tube.

Imaging was performed using a Jtomo microtomograph built in‐house at the University of Jyväskylä, with an isotropic voxel size of 6.7 μm. The X‐ray tube operated at 60 kV and 133 μA without filtration. A total of 6000 projections (2940 × 2304 pixels, 1500 ms exposure) were acquired during a continuous 360° rotation, resulting in a total scan time of around 3 h, including beam stabilization and reference image acquisition. The projections were corrected for beam hardening and ring artifacts and subsequently reconstructed into 3D μCT images using the filtered back‐projection algorithm. Each image was manually reoriented in 3D space to match the coordinate system of the brain atlas (Paxinos and Watson 1998), after which the electrode tip positions were identified from cross‐sectional slices extracted from the μCT images, based on residual tracks left in the tissue. Representative cross‐sectional slices showing the placement of electrodes in the CA1 and in the DG, along with the corresponding raw LFP signals, are depicted in Figure S2.

### 2.7 Data Analysis

#### 2.7.1 Hippocampal Electrophysiology

All analyses were performed off‐line with MATLAB using custom scripts. For all analyses, data were low‐pass filtered to avoid aliasing and resampled to 2 kHz. The timing of SPW‐Rs, theta epochs, bursts of fast and slow gamma, and DSs was extracted from the LFPs similar to (Lensu et al. 2021). Recordings from urethane‐anesthetized rats were processed from 60 min onward to allow the probes to settle in. Recordings from chronically implanted animals were processed such that DSs and SPW‐Rs were only looked for when the rat was immobile. Specifically, the average of the absolute accelerometer signal amplitude had to remain below a set threshold value (1000 arbitrary units) during a 300‐ms period immediately preceding the analysis window.

In short, SPW‐Rs were detected from a signal assumed to originate in the CA1 pyramidal layer. The signal was band‐pass filtered (120–230 Hz) and the mean (M) and SD of the filtered signal derived. Peaks in the filtered signal above M + 4 × SD for at least 20 ms were treated as putative SPW‐Rs when analyzing the urethane‐anesthesia recordings. For the chronic recordings, the threshold was set at M + 5 × SD to avoid detecting artifacts from muscle activity in the head. Only events at least 50 ms apart were treated as separate SPW‐Rs. A similar approach was used to detect fast (80–140 Hz) and slow (25–80 Hz) gamma bursts from DG LFPs (Bragin, Jando, Nadasdy, Hetke, et al. 1995; Colgin 2015) during acute recordings. The threshold for both types of gamma bursts was M + 2.5 × SD. The burst had to last at least 40 ms and be separated from another by at least 100 ms. DSs were detected from DG LFPs based on measures from two consecutive 10‐ms (sliding) windows. To qualify as a DS, the signal had to increase in amplitude from one window to another by a value greater than the M + 4 × SD when analyzing the acute urethane‐anesthesia recordings. For the chronic recordings, the limit was set at M + 3 × SD to catch all DSs. From the acute urethane‐anesthesia recordings, theta epochs were detected from the molecular layer signal, based on the relative power of the theta oscillation compared to the combined power of other slow oscillations (3.4–8.2 Hz/0.5–20 Hz). A 500‐ms epoch was considered theta if the ratio was above 80. For both visualization and further analysis, the SPW‐Rs, theta, gamma, and fast gamma epochs were aligned based on the largest negative peak and phase of the oscillation, and DSs based on the positive peak. The phase was derived from the band‐pass filtered signal by means of first calculating the Hilbert transform and then using the angle function in MATLAB.

Note that for the urethane recordings, the identified theta epochs were later used to categorize other events (DSs, SPW‐Rs) as taking place in a REM‐like or a NREM‐like state. If there was a theta epoch detected within a 5‐s period leading up to the event, it was classified as taking place during a REM‐like state. Otherwise, the event was categorized as taking place during a NREM‐like state.

**FIGURE 1:**
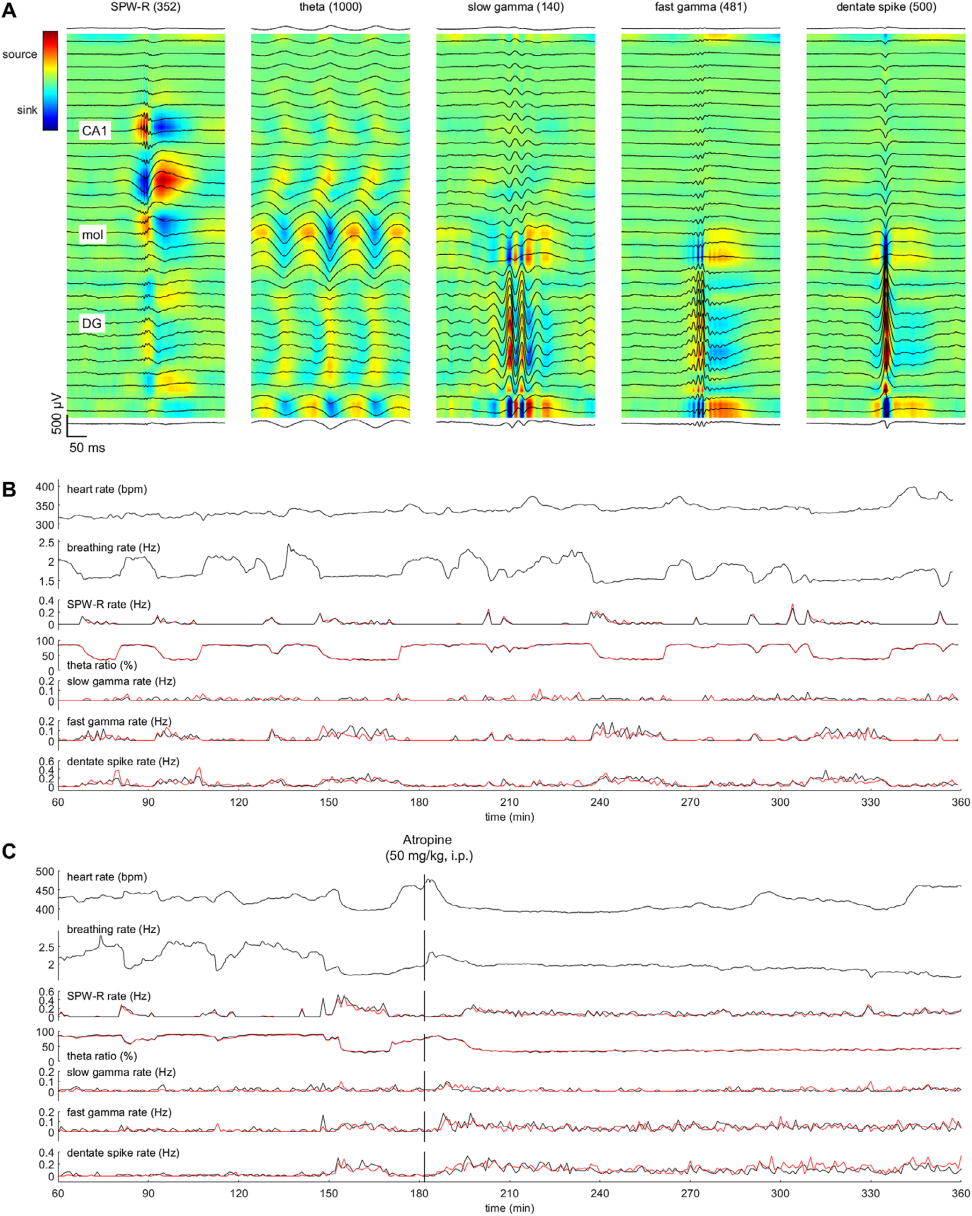
Hippocampal oscillations were recorded together with electrocardiogram and breathing for several hours under urethane (1.3 g/kg) anesthesia in adult male Sprague–Dawley rats. (A) Sharp‐wave ripples (SPW‐Rs), theta, and gamma (slow: 25–80 Hz; fast: 80–140 Hz) and dentate spikes were detected offline from local‐field potentials (LFPs). Average LFPs along the 32 electrode contacts are plotted in black, and the underlying color image reflects current source density values. Example from one rat. (B) Timing of events in the same rat, as in panel (A). Changes in signals across time illustrate fluctuation between REM‐like (high theta ratio, low SPW‐R rate) and NREM‐like states. (C) Timing of events from a different rat in which atropine (50 mg/kg) was injected i.p. after ~3 h of recording. Note that after the atropine injection, the rate (Hz) of SPW‐Rs or DSs is within the same range as before, but the animal remains in a NREM‐like state. In (B) and (C), red refers to events detected in the right dorsal hippocampus; black refers to events detected in the left dorsal hippocampus.

From all suitable epochs of linear probe recordings with an event detected, we calculated a CSD map and plotted that together with the averaged LFPs to visualize the data for each subject and event type. Examples of hippocampal electrophysiological oscillations in a representative rat under urethane anesthesia are illustrated in Figure 1A. Examples of event timing in a representative rat under urethane are shown in Figure 1B and in a representative rat under urethane and atropine in Figure 1C.

#### 2.7.2 Bilateral Events and Phase Synchrony

An event was determined bilateral if it occurred in one hemisphere (seed) and within a ±50 ms time window of that event peak/center, there was also an event in the contralateral hemisphere. For statistical descriptives and testing of the data recorded acutely under urethane‐anesthesia with the linear probes, a common measure (% of bilateral events) was calculated, that is, laterality was determined using left hemisphere as the seed and using right hemisphere as the seed and then an average of the two results was calculated. For the data from chronically implanted rats, the seed was determined as the hemisphere with the lower number of events of a certain type to avoid underestimating synchrony between hemispheres due to possibly nonoptimal electrode depth (see Figure 5A,B).

From the linear probe data recorded under urethane‐anesthesia, to examine the phase synchrony of right and left hippocampus during bilateral SPW‐Rs, LFPs from the CA1 pyramidal layer electrode in each hemisphere were band‐pass filtered either at the sharp‐wave band (3–12 Hz) or at the ripple band (120–230 Hz), and a standardized value indicating phase synchrony was calculated based on (Palva et al. 2005) as described in (Wikgren et al. 2010). Values greater than 1 indicate statistically significant phase synchronization at the 0.05 alpha level. Statistical significance at the group level at each time point was further determined using the binomial test and assuming a 50% chance of obtaining phase synchrony values above 1.

#### 2.7.3 SPW‐R Bouts in Urethane Anesthesia

SPW‐R bouts were determined from the linear probe LFPs from both hippocampi at once: A bout consisted of at least 10 SPW‐Rs in the left and/or right hippocampus with a maximum interevent interval of 30 s. The start of the bout was set as the peak time of the first SPW‐R included in that bout. A gap longer than 30 s terminated the bout, and the stop was set at the peak time of the last SPW‐R included in that bout. For statistical testing, the respiration rate as well as heart rate were determined from a 1‐min period prior to and a 1‐min period immediately after the start of each bout and averaged over rats and conditions (urethane vs. urethane + atropine).

### 2.8 Respiration and Cardiac Cycle

Note that respiration and ECG were only recorded from rats recorded acutely under urethane anesthesia but not from chronically implanted rats. Respiration rate was determined in Hz based on the difference (ms) in piezo signal peaks. Respiration phase was determined from the piezo signal using the Hilbert transformation and the angle function in MATLAB. Lungs were considered full when the piezo signal reached peak (at 0 rad) and lungs were considered empty when the piezo signal reached trough (−π/π). The piezo signal phase information was binned into 10 equal‐sized bins from −π to π. Each hippocampal electrophysiological event was assigned to its corresponding piezo signal bin. Because inspiration and expiration are usually not equal in duration, the occurrence rates (% of all events) per piezo signal phase bin were corrected by the proportion (%) of time spent in each phase bin to arrive at an unbiased distribution.

The ECG signal was analyzed for R‐peaks and heart rate determined based on interpeak interval. To determine phases of the cardiac cycle, the time between R‐peaks was divided into 10 bins of equal duration. Then, each hippocampal electrophysiological event was assigned to its corresponding ECG bin and the distribution examined statistically (see below).

For studying the effect of breathing rate on lateralization of DSs and SPW‐Rs, the mean and SD of breathing rate (Hz) were determined for each animal. Then, events were split into two categories based on a threshold of M—1.5 × SD and the breathing rate during a 5‐s period leading up to each event. The proportion (%) of bilateral events of all events taking place during slow breathing was compared to that during higher‐frequency breathing.

### 2.9 Statistical Analyses

IBM SPSS (IBM, Armonk, NY, USA), GraphPad Prism (GraphPad Software, Boston, MA, USA), and MATLAB were used for all statistical analyses. Paired samples *t* tests were used for comparisons within subjects. Phase preference was analyzed using the Circular Statistics Toolbox for MATLAB (Berens 2009). Specifically, we used the functions “circ_mean” and “circ_rtest” to determine whether hippocampal oscillations took place preferably at a certain phase of the respiration or cardiac cycle in each rat. The first function gives the mean direction or angle of the resultant vector, and the second function tests for nonuniformity of occurrence. Data from a rat were included in the analysis if at least 50 hippocampal oscillatory events under examination were detected.

## 3 Results

### 3.1 Hippocampal Oscillations Under Urethane Anesthesia Were Connected to Respiration to a Variable Degree

**FIGURE 2:**
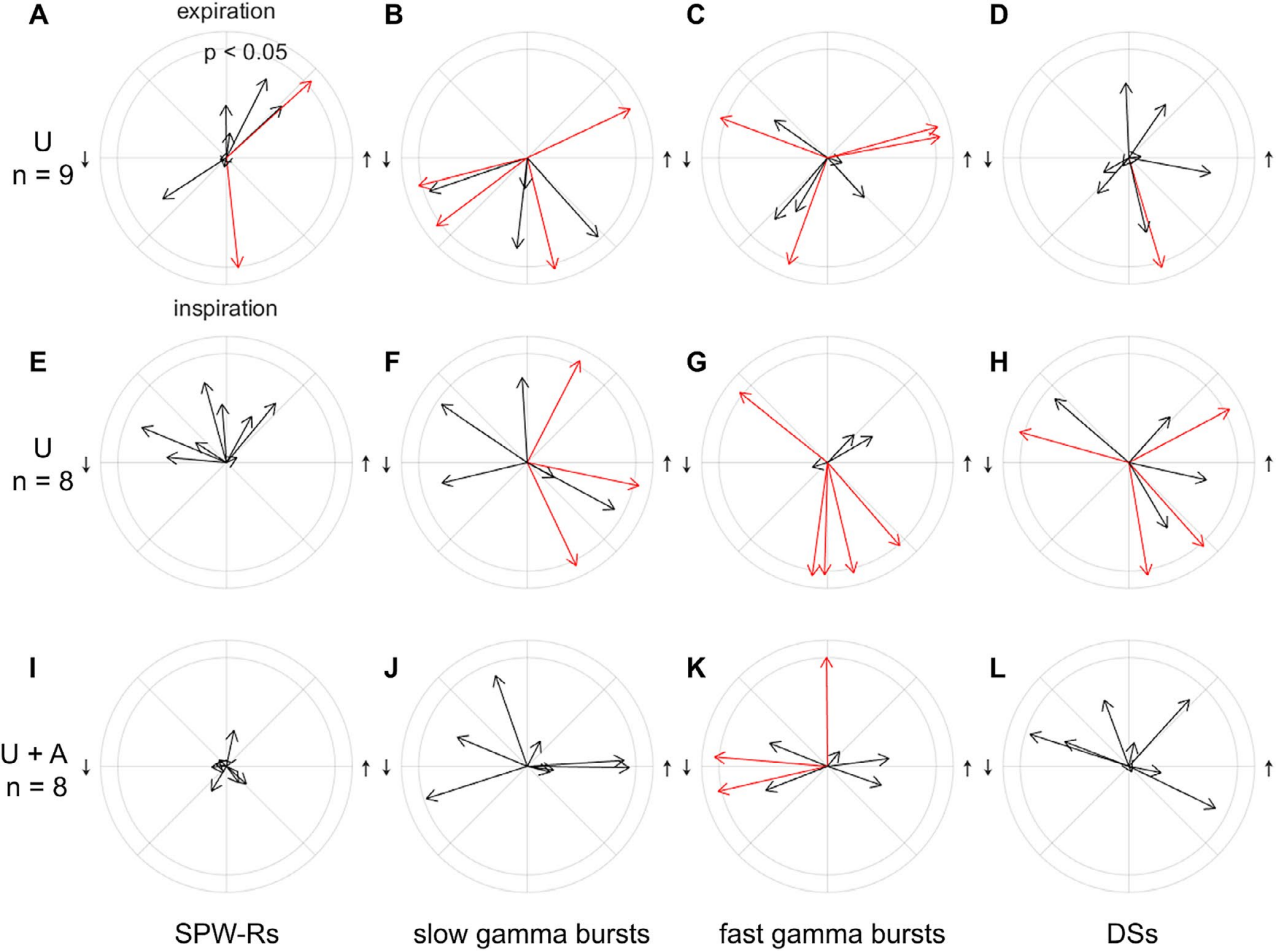
Connection of hippocampal oscillations to respiration. Distribution of (A) SPW‐Rs, (B) slow gamma bursts, (C) fast gamma bursts, and (D) DSs across the respiration cycle. Data from the first group (*n* = 9) of rats under urethane (U) anesthesia. (E–H) Similar plots for the second group of animals (*n* = 8), under urethane only (U), and (I–L) under urethane and atropine (U + A). Statistical significance of circular statistics is reflected by the color of the arrow: Red, *p* < 0.050.

We sought to find out if hippocampal oscillations link to certain phases of the cardiac and respiration cycles (Karalis and Sirota 2022; Liu et al. 2017) (see Figure 2). In the group (*n* = 9) anesthetized with urethane, heart rate was 363 (26) bpm, breathing rate was 1.71 (0.13) Hz, and the overall theta ratio was 69 (7)%. Please see Figure 1B for an example of changes across time in one representative rat. According to circular statistics (Berens 2009), SPW‐Rs were statistically significantly biased to a certain phase of respiration in 2/9 rats under urethane anesthesia (Figure 2A): in one rat the preferred phase was inspiration (−1.46 rad, z = 3.20, *p* = 0.040) and in the other it was expiration (0.74 rad, z = 5.73, *p* = 0.003). Slow gamma bursts were statistically significantly biased to inspiration in four rats (−2.89–0.44 rad, z = 3.88–13.84, *p* = 0.000–0.020, Figure 2B). Fast gamma bursts were also time‐locked to respiration in four rats, but the preferred respiration phase was more varied: −1.92 to 2.78 rad (z = 4.38–9.72, *p* = 0.000–0.012, Figure 2C). DSs were statistically significantly linked to inspiration, but only in one rat (−1.28 rad, z = 7.43, *p* = 0.001, Figure 2D). To summarize, hippocampal oscillations were linked to respiration phase, but there was considerable variation in the magnitude of phase‐locking and the exact preferred phase. Cardiac cycle phase was not associated with occurrences of any of the hippocampal oscillations studied (data not shown).

In the group (*n* = 8) first anesthetized with urethane and then administered atropine (50 mg/kg, i.p.) ~3 h into the recording, heart rate was on average 395 (35) bpm before and 404 (22) bpm after the atropine injection, *t* (7) = 0.88, *p* = 0.407. Breathing rate was 1.97 (0.19) Hz before and 2.00 (0.12) Hz after the atropine injection, *t* (7) = 0.61, *p* = 0.558. As expected, there was a statistically significant drop in theta ratio from 60 (18)% prior to atropine injection to 44 (4)% after atropine injection, *t* (7) = 2.90, *p* = 0.023. Please see Figure 1C for an example of fluctuations across time in one rat. SPW‐Rs were not statistically significantly biased to a certain phase of respiration in any of the rats before (z = 0.11–1.62, *p* = 0.198–0.898, Figure 2E) or after (z = 0.00–0.39, *p* = 0.679–0.998, Figure 2I) the atropine injection. Slow gamma bursts were statistically significantly biased to inspiration in three rats before (−1.12 to 1.09 rad, z = 9.22–15.18, *p* < 0.001, Figure 2F) and in none of the rats after (z = 0.23–2.63, *p* = 0.071–0.796, Figure 2J) the atropine injection. Fast gamma bursts were time‐locked to respiration in five rats prior to atropine injection (z = 3.35–28.94, *p* = 0.000–0.035, Figure 2G); the preferred respiration phase was varied (−1.71 to 2.47 rad). After the atropine injection, fast gamma bursts were still time‐locked to variable phases of respiration in three rats (−2.92 to 3.06 rad, z = 3.05–4.74, *p* = 0.009–0.047, Figure 2K). DSs were statistically significantly linked to a certain phase of respiration in four rats prior to atropine (−1.41 to 2.87 rad, z = 4.15–9.68, *p* = 0.0001–0.016, Figure 2H), while after atropine injection this link was absent in all rats (z = 0.06–2.41, *p* = 0.090–0.943, Figure 2L). Cardiac cycle phase was not associated with occurrences of any of the hippocampal oscillations studied (data not shown). To summarize, as in the previous group of rats, under urethane only, also under urethane and atropine, hippocampal oscillatory phenomena connected to respiration phase but there was abundant variation in the magnitude of phase‐locking and the exact preferred phase.

### 3.2 A Decrease in Respiration Rate Preceded SPW‐R Bout Onset Regardless of Cholinergic Tone Under Urethane Anesthesia

**FIGURE 3:**
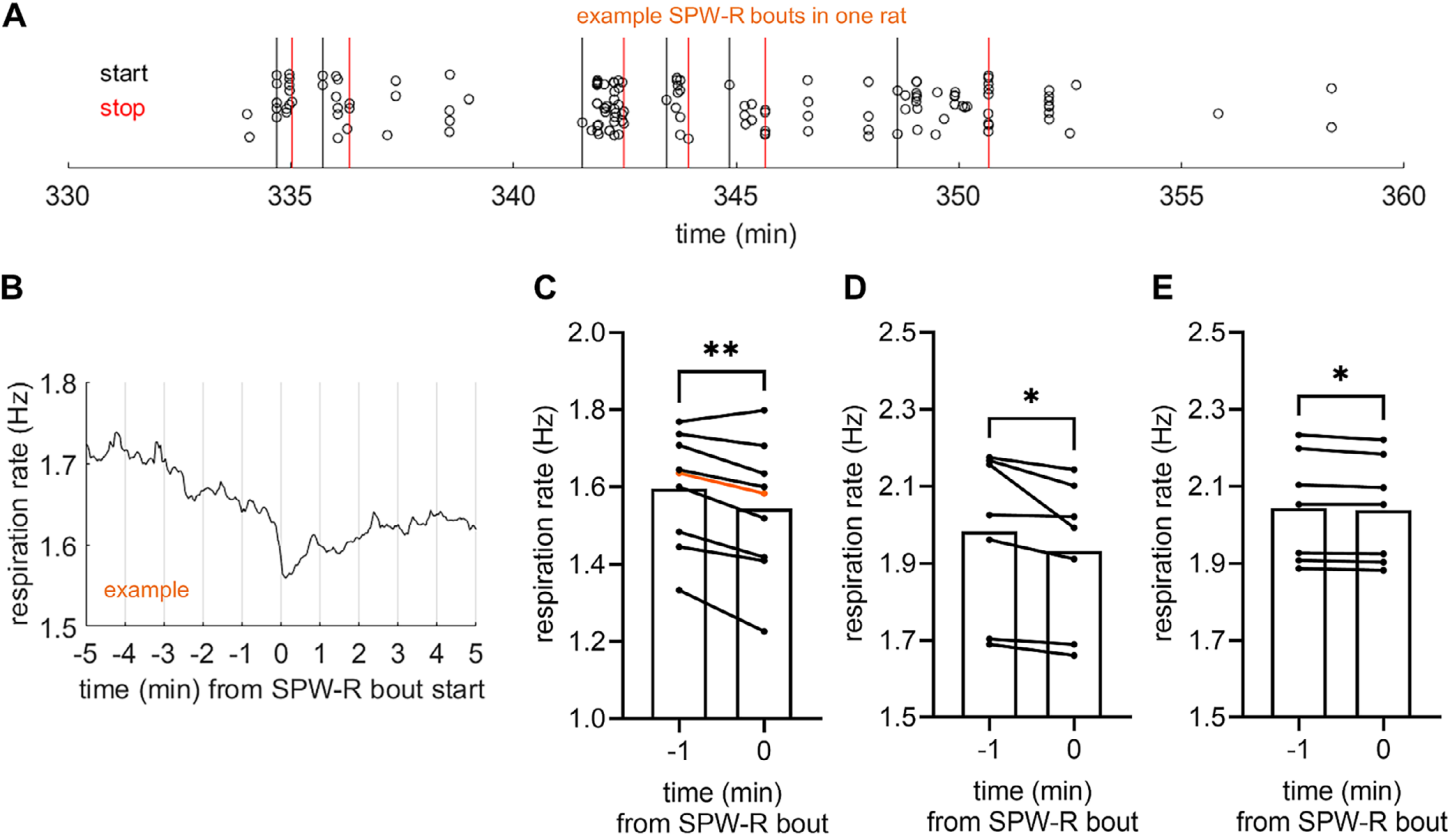
SPW‐R bouts were paralleled by a decrease in respiration rate under urethane anesthesia. (A) Example of SPW‐R bout timing in one rat. Each open circle corresponds to an individual SPW‐R. Markers are jittered along the y‐axis to reduce overlap and allow better visualization. (B) Example of respiration rate around SPW‐R bout start in the same rat. (C) In rats under urethane anesthesia (*n* = 9), respiration rate was lower during than before SPW‐R bouts. Data from B is shown in orange. (D) In the second group of rats (*n* = 8), respiration rate was lower during than before SPW‐R bouts both under urethane only and (E) under urethane and atropine. Asterisks refer to results of paired samples *t* test: **p < 0.010, *p < 0.050.

In addition to quickly alternating phases in respiration and cardiac cycle, we thought it likely that the overall state of the body might be linked to hippocampal function as suggested by earlier studies (Pagliardini et al. 2012). Inspecting the data by eye (see Figure 1B), we quickly noticed a decreasing trend in respiration rate related to the occurrence of SPW‐Rs. Thus, we determined start times for SPW‐R bouts and derived the changes in respiration rate and heart rate around that time point (see Figure 3A and Methods). Paired samples *t* test confirmed that while there was no change in heart rate (*t* [8] = 0.21, *p* = 0.842), respiration rate decreased statistically significantly from that measured during a 1‐min period prior to a SPW‐R bout to that measured immediately after the onset of the bout: *t* (8) = 3.97, *p* = 0.004 (Figure 3B,C). In the group (*n* = 8) administered atropine (50 mg/kg, i.p.) ~3 h into the recording, SPW‐R bouts were detected in 7/8 rats and were preceded by a decrease in breathing rate both before (*t* [6] = 2.52, *p* = 0.045; Figure 3D) and after (*t* [6] = 3.14, *p* = 0.020, Figure 3E) the atropine injection. That is, the lowered cholinergic tone did not alter the relationship between respiration rate and SPW‐R occurrence.

### 3.3 Laterality of DSs and SPW‐Rs Varied Across States Under Urethane Anesthesia

**FIGURE 4:**
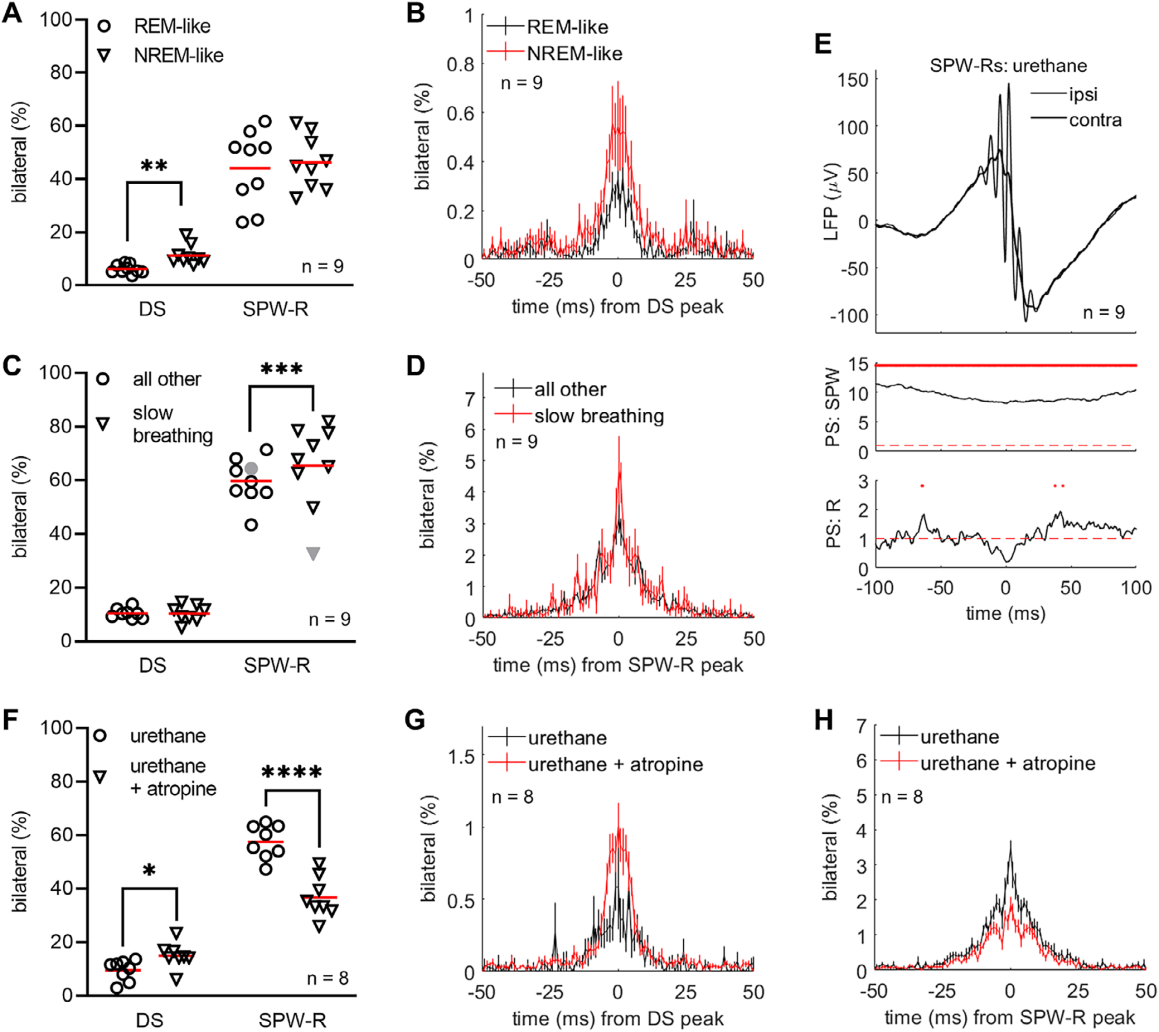
Lateralization of dentate spikes (DS) and sharp‐wave ripples (SPW‐R) varied across states under urethane anesthesia. All events in one hemisphere accompanied by an event in the contralateral hemisphere within a ±50 ms window were deemed bilateral. (A and B) The proportion of bilateral DSs and SPW‐Rs during REM‐like and NREM‐like states in 9 rats under urethane anesthesia. (C and D) The proportion of bilateral DSs and SPW‐Rs during slow breathing (M—1.5 × SD) and at all other times. Note that one rat had very few DSs during slow breathing (omitted) while another (in gray) displayed a pattern in SPW‐R laterality during slow vs. other breathing opposite to all other rats and was excluded from statistical testing. (E) Phase synchrony (PS) of hippocampal CA1 pyramidal cell layer LFPs during bilateral SPW‐Rs (top) was interrogated at frequency bands corresponding to the sharp wave (SPW, 3–12 Hz, middle) and the ripple (R, 120–230 Hz, bottom). Phase synchrony between the left and right hippocampi was statistically significant (standardized values above 1; red dotted line illustrates the threshold) at the SPW band, but low at the ripple band. Red dots above the PS curves indicate statistically significant PS at the group level according to binomial test. (F–H) The proportion of bilateral DSs and SPW‐Rs in a separate group of eight rats recorded under urethane anesthesia and later injected with 50 mg/kg (i.p.) atropine. Asterisks refer to results of paired samples *t* test: *p < 0.050, **p < 0.010, ***p < 0.001, ****p < 0.0001.

In addition to the connection of hippocampal oscillations to bodily signals, we were also interested in the interhemispheric synchrony of DSs and SPW‐Rs and how this is affected by cholinergic tone (Gedankien et al. 2023). In urethane‐anesthetized adult male rats (*n* = 9, Figure 4A–E), most hippocampal DSs were restricted to one hemisphere: Only on average 6 (2)% of DSs were bilateral during a REM‐like state and 11 (4)% during a NREM‐like state, as evaluated based on the presence or absence of theta epochs during a 5‐s period leading up to the DS (see Methods) (Figure 4A,B). According to a paired samples *t* test, there was a statistically significant difference in DS laterality between REM‐like and NREM‐like states: *t* (8) = 3.69, *p* = 0.006. On the contrary, SPW‐Rs were equally bilateral during a REM‐like state (44 [14] %) and during a NREM‐like state (46 [10] %): *t* (8) = 0.36, *p* = 0.727. Inspired by the fact that fluctuations in cholinergic tone are reflected in respiration (Pagliardini et al. 2012), we also studied the effect of breathing rate (*n* = 9, 1.74 [0.13] Hz) on the laterality of DSs and SPW‐Rs (Figure 4C,D). Comparing the proportion of bilateral DSs during slow breathing, at a rate of 1.5 SDs below average, to that of all other DSs revealed no statistically significant difference (*n* = 8, 10 [2] % vs. 10 [3] %, respectively; *t* [7] = 0.07, *p* = 0.946). Note that one rat had to be excluded as very few DSs took place during slow breathing. The proportion of bilateral SPW‐Rs was higher during slow breathing compared to that measured during breathing rates above the threshold in eight out of nine rats. The difference was statistically significant (*n* = 8, 59 [9] % vs. 69 [11] %, respectively; *t* [7] = 6.38, *p* = 0.0004). In Figure 4E, the phase synchrony (standardized units) of left and right CA1 pyramidal layer LFPs during bilateral SPW‐Rs under urethane anesthesia (*n* = 9 rats) is visualized. The synchrony is statistically significant, that is, above 1, for the sharp‐wave frequency band but not for the ripple band. There were no apparent differences between REM‐like and NREM‐like states in phase synchrony (data not shown).

In a different group of rats (*n* = 8, Figure 4F–H), first anesthetized with urethane and later injected with atropine (50 mg/kg, i.p.), we explored whether directly manipulating cholinergic tone has any effect on the lateralization of DSs and SPW‐Rs. There were no apparent effects of atropine on the phase synchrony between the left and right CA1 pyramidal cell layer LFPs during SPW‐Rs (data not shown). Under urethane only, 10 (4)% of DSs and 58 (6)% of SPW‐Rs were bilateral. Under urethane and atropine, the corresponding values were 15 (5)% and 37 (8)%, respectively. Paired samples *t* tests indicated that atropine increased the proportion of bilateral DSs, *t* (7) = 3.35, *p* = 0.012, and decreased that of bilateral SPW‐Rs, *t* (7) = 10.20, *p* < 0.0001.

To summarize, DSs originating in the EC were more often bilateral when the hippocampal theta ratio was low (NREM‐like state) than when it was high (REM‐like state). SPW‐Rs were more often bilateral during slow breathing compared to faster breathing. Low cholinergic tone and inhibition of parasympathetic effects due to atropine increased the proportion of bilateral DSs and decreased that of bilateral SPW‐Rs. Sharp‐waves were consistently phase‐synchronized between hemispheres during bilateral SPW‐Rs, while ripples were not, and no differences in phase synchrony were evident when comparisons were made based on ongoing brain or bodily state.

### 3.4 DSs and SPW‐Rs Were Mostly Bilateral in Awake Rest and Natural Sleep

**FIGURE 5:**
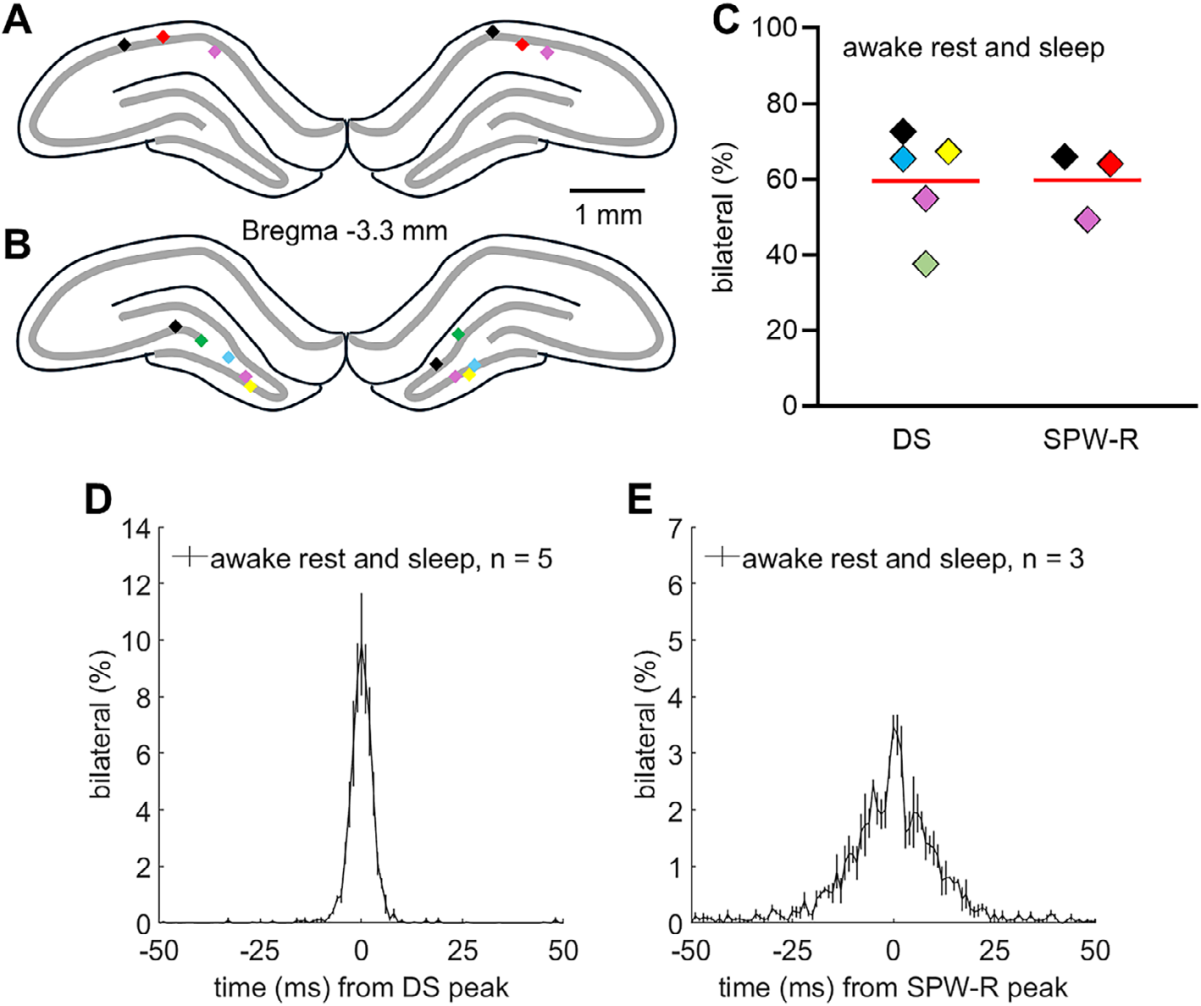
DSs and SPW‐Rs were mostly bilateral in awake rest and natural sleep. (A and B) Sharp‐wave ripples were bilaterally captured in three rats (A) and dentate spikes in five rats (B). Each color represents one rat. (C) Both types of hippocampal events were bilateral ~60% of the time. Red line indicates mean. (D and E) The timing of the corresponding event peak in the hemisphere opposite to that used as seed. Group average is plotted and the SEM indicated with vertical lines.

To confirm whether urethane anesthesia might be the main source of low interhemispheric synchrony of especially DSs, we conducted analyses of LFP data obtained from single monopolar electrodes chronically implanted in the dorsal hippocampi in adult female rats (see Figures S2 and 5A,B). Altogether, six rats were recorded with electrodes bilaterally placed in the CA1 in three rats and in the DG/hilus in five rats. Up to four ~2‐h recordings per rat were used for analysis. Using the hemisphere with fewer events detected as the seed (to avoid underestimating synchrony), we found that 60 (9)% of SPW‐Rs were bilateral and 60 (14)% of DSs were bilateral (see Figure 5C–E below) during awake rest or sleep, that is, when the accelerometer signal indicated immobility.

## 4 Discussion

We studied how the occurrence of hippocampal electrophysiological oscillations relates to respiration, heartbeat, and cholinergic tone in urethane‐anesthetized rats. As expected (Karalis and Sirota 2022), respiration phase was associated with SPW‐Rs, gamma oscillation bursts, and DSs. However, there was considerable interindividual variation, and atropine attenuated the link. We found no consistent connection between cardiac cycle phase and hippocampal oscillations. A decrease in respiration rate preceded the onset of SPW‐R bursts, both under urethane only and under urethane and atropine, while we found no link between heart rate and the occurrence of SPW‐Rs. In line with earlier findings (Hernández‐Recio et al. 2023; Lehtonen et al. 2022), DSs were mostly unilateral, whereas SPW‐Rs took place bilaterally roughly half of the time under urethane anesthesia. The interhemispheric synchrony of DSs was higher during the NREM‐like state and under the influence of atropine, while the opposite was true for SPW‐Rs. Consistently, slow breathing suggestive of high parasympathetic drive was associated with increased hemispheric synchrony of SPW‐Rs. In awake rest or natural sleep, ~60% of both SPW‐Rs and DSs were bilateral. These results together suggest a link between the overall brain and bodily states and a specific link between respiration and hippocampal electrophysiological events thought to support memory consolidation during rest and sleep. Finally, our results highlight the differences in brain electrophysiology between urethane anesthesia and natural rest/sleep.

Our current results indicate that hippocampal oscillations might synchronize to breathing in urethane‐anesthetized rats, but there was considerable intersubject variation (Figure 2). Further, we found no link between the cardiac cycle phase and hippocampal oscillations. In head‐fixed, naturally sleeping mice, Karalis and Sirota (2022) report that most hippocampal DG and CA1 principal neurons fire, and most DSs and SPW‐Rs take place, after inspiration. Further, they report sinks in the outer and middle molecular layer of the DG, respectively, tied to inspiration, suggesting input from layer II neurons in the lateral and medial EC is orchestrated by the breathing rhythm (Karalis and Sirota 2022). It is possible that the connection between breathing cycle phases and brain function is species‐specific and does not generalize between mice and rats. Another possibility remains that the use of urethane in our current study might have dampened the connection between breathing and hippocampal activity.

However, there seems to be a link between the overall state of the animal and hippocampal function: We report a decrease in respiration rate leading up to the onset of SPW‐R bouts (Figure 3) under urethane anesthesia. This link between a decrease in breathing rate and SPW‐R bout onset was not abolished by atropine, which induced a steady NREM‐like state where SPW‐Rs took place often and the changes in respiration rate overall were small. Consistent with the known effects of atropine, SPW‐Rs in mice have been reported to occur in increasing numbers when cholinergic signaling is endogenously decreased during NREM (Zhang et al. 2021). Further, alternation between REM‐like and NREM‐like states in urethane‐anesthetized rats associates with respective increases and decreases in breathing rate (Pagliardini et al. 2012). Taken together, it seems that variation in cholinergic or parasympathetic tone affecting respiration rate could also affect the occurrence of SPW‐Rs. Whether there is a causal link from respiration to hippocampal oscillations remains to be investigated. Our current observations do hint that regulating respiration rate might offer a way to also manipulate brain activity related to memory consolidation.

One way to test the idea stated above would be to directly manipulate the activity of the pre‐Bötzinger complex (preBötC) pacemaker cells (for review see [Ramirez et al. 2011]): The firing of preBötC neurons initiates inspiration, activates sympathetic outflow, and depresses parasympathetic output (Menuet et al. 2020). The preBötC neurons receive input from the paraventricular nucleus of the hypothalamus (PVN) (Mack et al. 2007), which is important for controlling, for example, feeding and stress responses via the hypothalamic–pituitary–adrenal axis (Qin et al. 2018). Interestingly, the PVN is also connected to the hippocampus (Buijs 1978). Further, the PVN receives input from the thalamic paraventricular nucleus (PVT) (Ferguson et al. 1984), which also projects to the hippocampus (and the medial prefrontal cortex) (Viena et al. 2022). On the contrary, the preBötC neurons themselves only send sparse efferent connections to the forebrain, and even these are restricted to nuclei in the thalamus and hypothalamus (Yang and Feldman 2018). They do, however, project densely to other brain stem regions involved in the control of breathing, including the parahypoglossal region of the medulla and the nucleus of the solitary tract (NTS) (Tan et al. 2010; Yang and Feldman 2018). The NTS is connected to the PVN (Affleck et al. 2012) and to the locus coeruleus (LC), which then connects to the hippocampus to facilitate learning (Mello‐Carpes and Izquierdo 2013; Tsetsenis et al. 2022). In fact, electrical stimulation of the LC during SPW‐Rs (Novitskaya et al. 2016) or pharmacological manipulation of norepinephrine activity (and hence SPW‐Rs) after training (Durán et al. 2023) both hinder learning. To summarize, while there is no report of a direct anatomical link between the preBötC and the hippocampus, the various indirect bidirectional connections described above might help explain how respiration could link to hippocampal activity. Clearly, more studies are needed to identify the mechanisms underlying the connection between respiratory and forebrain function.

A previous study in urethane‐anesthetized rats reported high bilateral synchrony for CA1 LFPs but lower synchrony for DG events (Hernández‐Recio et al. 2023). In our current data from urethane‐anesthetized rats, SPW‐Rs were detected in both hippocampi within 50 ms of each other roughly half the time (Figure 4). The proportion of bilateral SPW‐Rs was similar during awake rest or sleep (Figure 5C). In general, the proportion of bilateral SPW‐Rs both under urethane anesthesia and in natural rest or sleep in our current study seems higher than that reported earlier during natural sleep in Sprague–Dawley rats (Villalobos et al. 2017) but resembles that reported in mice (Farrell et al. 2024). Atropine significantly decreased the proportion of bilateral SPW‐Rs in our rats under urethane anesthesia. Consistent, slow breathing suggestive of high parasympathetic drive during the NREM‐like state (Pagliardini et al. 2012) was associated with increased hemispheric synchrony of SPW‐Rs in the majority of rats. To recap, our results suggest that muscarinic receptor function and parasympathetic nervous system activation regulate synchronization of SPW‐Rs in the left and right hippocampus. Our results on phase synchrony indicated significant bilateral synchrony of sharp waves but not of ripples. This is in line with earlier findings: The occurrence of SPW‐Rs is correlated with brain‐wide activity patterns (Nitzan et al. 2022), and the EC seems to control SPW‐R generation (Zutshi and Buzsáki 2023). However, the pyramidal cell firing patterns are assumed to originate from the CA3 (Ecker et al. 2022).

In contrast to SPW‐Rs, DSs during urethane anesthesia were almost always unilateral as reported earlier (Lehtonen et al. 2022) (but see in mice: [Farrell et al. 2024]) but bilateral to a similar degree as SPW‐Rs in the absence of anesthesia. Thus, it seems that urethane dissociates inputs from the left and right EC to the corresponding hippocampus. This is in line with a previous report of desynchronization of the left and right neocortex in urethane‐anesthetized rats, especially involving higher frequency activity in the S1 region during a NREM‐like state (Mondino et al. 2024). It is assumed that input from the EC to the DG responsible for DSs (Bragin, Jando, Nadasdy, van Landeghem, et al. 1995) arrives via the perforant path, a mainly unilaterally targeting pathway (Golarai and Sutula 1996). During bilateral DSs, signals from the left and right EC would need to be sent at the same time, or alternatively, unilateral EC signals would need to reach the contralateral DG via the perforant pathway or indirectly via the association pathway or the hippocampal commissure, both originating in the hilus (Swanson et al. 1981). Interestingly, during the NREM‐like state and under atropine, DSs were more often bilateral (although still mostly unilateral) in the urethane‐anesthetized rats. This could reflect synchronization of left and right EC activity or the potentiation of contralateral connections to the DG, or both. Further studies are needed to determine what other factors besides cholinergic signaling regulate interhemispheric synchronization. More importantly, in future studies, the effect of urethane on network activity and connectivity in the brain (see for example [Mondino et al. 2024] for more) needs to be considered when interpreting results.

To summarize, cholinergic tone seems to differently affect the synchronization of the left and right DG (DSs) compared to that of the left and right CA1 (SPW‐Rs). Obviously, in future studies, bilateral recordings should be favored over unilateral recordings to capture the full picture of hippocampal activity, as quite a large proportion of events might be unilateral. It will remain to be seen how the lateralization of SPW‐Rs and DSs is linked to memory formation in the hippocampus.

## Author Contributions

**Miriam S. Nokia:** conceptualization, investigation, formal analysis, writing – original draft, reviewing and editing, visualization. **Sanna Lensu:** conceptualization, investigation, writing – reviewing and editing. **Suvi‐Maaria Lehtonen:** investigation, writing – reviewing and editing. **Tero Harjupatana:** investigation, visualization, writing – original draft, reviewing and editing. **Markku Penttonen:** conceptualization, supervision, writing – reviewing and editing.

## Conflicts of Interest

The authors declare no conflicts of interest.

## Supporting information


**Figure S1:** Representative linear probe placement and raw LFP signals from the hippocampus during urethane anesthesia. Example of 32‐electrode linear probe placement (A) and raw local‐field potentials (B–D) recorded from the dorsal hippocampus. The electrode covered cell layers in the CA1 and in the dentate gyrus (DG). Signals used for event detection are plotted in black while all other signals are plotted in gray. In (B), the SPW‐R in the CA1 and the dentate spike in the DG are pointed by arrows. In (C) and (D), arrow points to gamma burst detected based on the DG signal. Theta is visible in the molecular layer signal (B–D).

**Figure S2:** Representative monopolar single electrode placement and raw LFP signals from the hippocampus during awake rest/sleep. (A) Example of electrode placement in the CA1, and (B and C) in the DG in one representative rat. (D) Local‐field potentials from a 2‐s period during which the rat was not moving. Red arrows point to dentate spikes and black arrows point to SPW‐Rs.

## Data Availability

The data that support the findings of this study are available from the corresponding author upon reasonable request.
