# Supplementary figures and images for: Lateralization of Hippocampal Dentate Spikes and Sharp‐Wave Ripples in Urethane Anesthetized Rats Depends on Cholinergic Tone
Source: Hippocampus. 2025 Sep 23;35(5):e70035. doi: 10.1002/hipo.70035 (PMC12455560; doi:10.1002/hipo.70035)

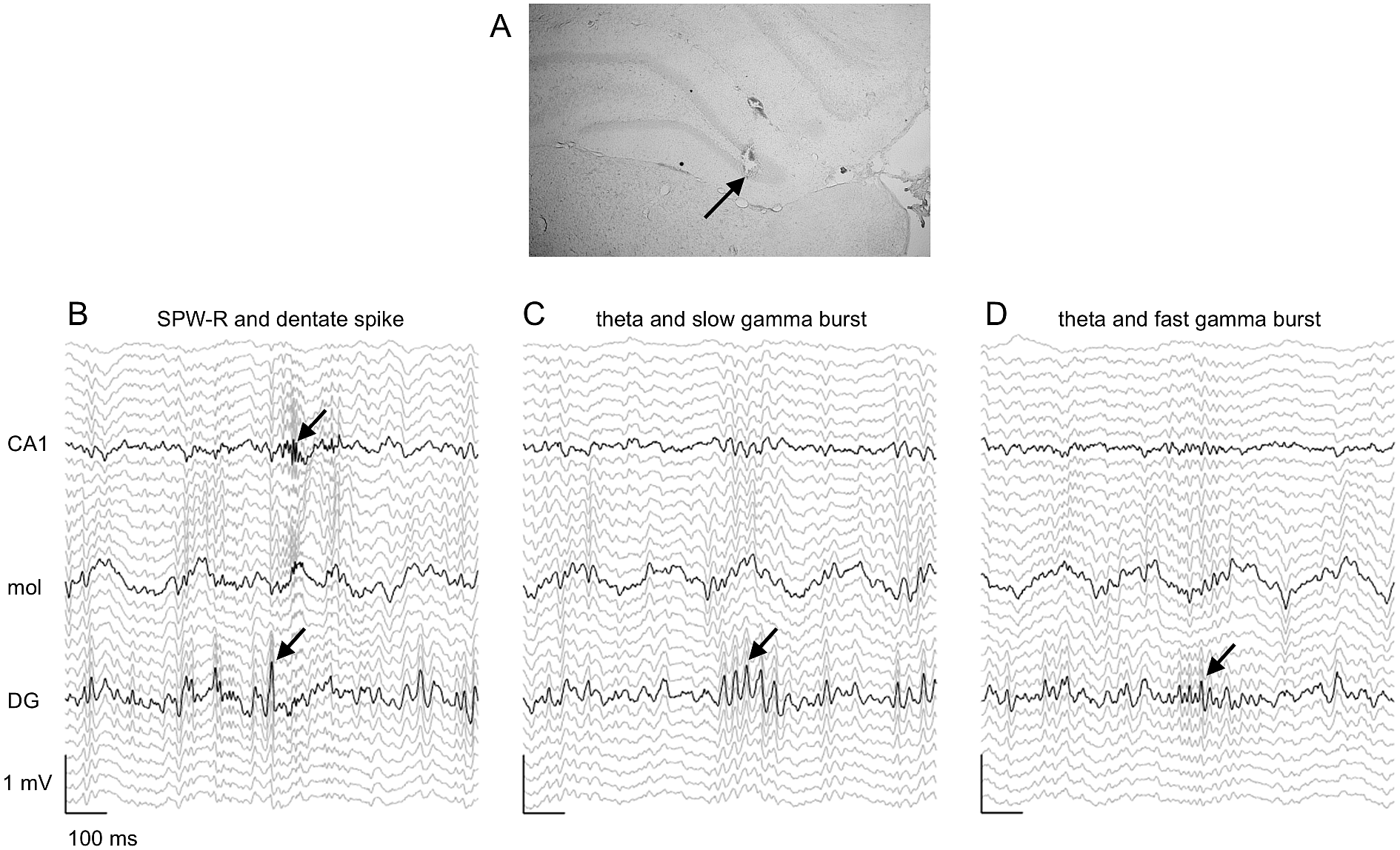

Supplement: Supplementary file 1 — Figure S1: Representative linear probe placement and raw LFP signals from the hippocampus during urethane anesthesia. Example of 32‐electrode linear probe placement (A) and raw local‐field potentials (B–D) recorded from the dorsal hippocampus. The electrode covered cell layers in the CA1 and in the dentate gyrus (DG). Signals used for event detection are plotted in black while all other signals are plotted in gray. In (B), the SPW‐R in the CA1 and the dentate spike in the DG are pointed by arrows. In (C) and (D), arrow points to gamma burst detected based on the DG signal. Theta is visible in the molecular layer signal (B–D). [file HIPO-35-0-s001.tif]

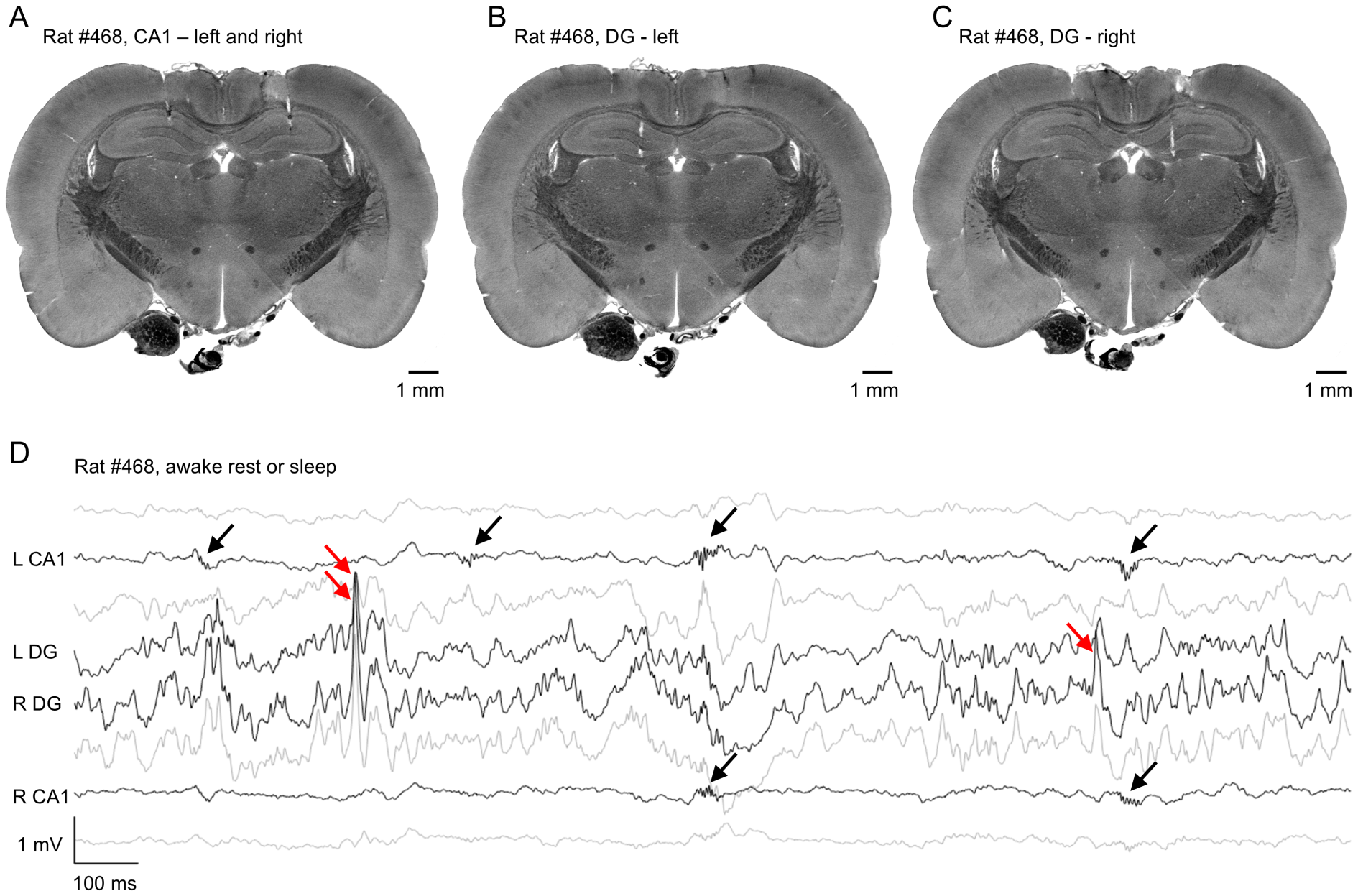

Supplement: Supplementary file 2 — Figure S2: Representative monopolar single electrode placement and raw LFP signals from the hippocampus during awake rest/sleep. (A) Example of electrode placement in the CA1, and (B and C) in the DG in one representative rat. (D) Local‐field potentials from a 2‐s period during which the rat was not moving. Red arrows point to dentate spikes and black arrows point to SPW‐Rs. [file HIPO-35-0-s002.tif]
